# High Glucose-induced transcriptomic changes in human trabecular meshwork cells

**DOI:** 10.1007/s11033-025-10525-z

**Published:** 2025-04-25

**Authors:** Shivendra Singh, Srimathi Raghavan, Niketa A. Patel, Avinash Soundararajan, Padmanabhan P. Pattabiraman

**Affiliations:** 1 Ophthalmology, Indiana University School of Medicine, Indianapolis, IN USA; 2 Research Service, JA Haley Veterans Hospital, Tampa, FL USA; 3 Stark Neuroscience Research Institute, Indiana University School of Medicine, Indianapolis, IN USA

**Keywords:** Glaucoma, Trabecular meshwork, Hyperglycemia, Oxidative stress, Fibrosis, Apoptosis, Autophagy

## Abstract

Glaucoma is a leading cause of irreversible blindness, often associated with elevated intraocular pressure (IOP) due to trabecular meshwork (TM) dysfunction. Diabetes mellitus (DM) is recognized as a significant risk factor for glaucoma; however, the molecular mechanisms through which hyperglycemia affects TM function remain unclear. This study investigated the impact of high glucose on gene expression in human TM (HTM) cells to uncover pathways that contribute to TM dysfunction and glaucoma pathogenesis under diabetic conditions. Primary HTM cells were cultured under normoglycemic (5.5 mM) and hyperglycemic (30 mM) conditions for seven days, followed by mRNA sequencing (mRNA-seq) to identify differentially expressed genes, with quantitative PCR (qPCR) used for confirmatory analysis. STRING network analysis was performed to predict potential interactions among upregulated and downregulated genes. mRNA-seq analysis revealed 25 significantly differentially expressed genes in high glucose conditions, including upregulated genes associated with oxidative stress, apoptosis, autophagy, immune response, and fibrosis. Notably, *TXNIP* gene was significantly upregulated, indicating increased oxidative stress and apoptosis in TM cells, while downregulation of autophagy-related genes, such as *HSPA6* and *LAMP3*, suggests compromised protein quality control. Immune response genes, including *CCL7* and *CHI3L1*, were upregulated, suggesting an inflammatory response to oxidative stress. Increased expression of fibrosis-related genes, such as *SNAI1*, *FGF7*, and *KRT19*, and an increase in ECM proteins like Collagen 1 and FN accumulation and fibril formation suggest increased fibrosis of TM in diabetic conditions, potentially elevating IOP. Metabolic changes in diabetic patients could therefore lead to TM dysfunction, impair aqueous humor outflow, and elevate IOP, thereby increasing glaucoma risk. Targeting oxidative stress and fibrosis pathways offers therapeutic strategies to mitigate glaucoma progression in diabetic populations.

## Introduction

Glaucoma is a group of eye diseases that lead to vision loss and eventual blindness by damaging the optic nerve in the eye [1]. This progressive eye disease is the leading cause of irreversible blindness and affects approximately 80 million people in the world [2, 3]. Glaucoma encompasses various subtypes, with the most common being primary open-angle glaucoma (POAG), normal-tension glaucoma (NTG), and angle-closure glaucoma (ACG) [1, 2, 4]. Except for NTG, glaucoma is typically characterized by elevated intraocular pressure (IOP), which remains the only modifiable risk factor [2, 4, 5]. The aqueous humor (AH) produced by the ciliary body flows into the anterior chamber, provides nutrients, and primarily exits through the conventional outflow pathway consisting of the trabecular meshwork (TM) and Schlemm’s canal (SC). The remaining AH exits through the uveoscleral outflow pathway [6, 7]. Increased resistance to AH flow is due to accumulation and reduced breakdown of extracellular matrix (ECM) components and increased actin cytoskeletal tension in the juxtacanalicular TM (JCT-TM) and SC regions. The AH buildup leading to IOP elevation results in glaucoma pathogenesis [8–11]. Aging, family history, hypertension, and diabetes are some of the proposed risk factors that contribute to the development of POAG [1, 12, 13].

Diabetes mellitus (DM) is a group of metabolic diseases characterized by impaired glucose regulation, affecting millions globally to the extent that it is considered a global pandemic [14]. Over 400 million individuals are estimated to have a DM diagnosis, with the number increasing annually [15]. Approximately 90% of cases are classified as Type 2 DM, which is largely hereditary, lifestyle, and/or environment-dependent [16, 17]. DM is associated with multiple microvascular complications throughout the body, including those affecting the vasculature of the eye [14, 16]. Studies indicate that elevated blood glucose in DM patients can permeate into the AH, with a direct correlation observed between blood glucose levels and glucose concentration in the AH [18, 19].

Diabetes is a significant risk factor for glaucoma worldwide [12, 20–28]. Diabetes and glaucoma are associated with metabolic dysregulation, which may be a result of TM dysfunction and ocular hypertension. Like glucose metabolism, lipid metabolism, including processes like de novo lipogenesis and cholesterol regulation, has been shown to influence cellular mechanisms critical for maintaining the structural integrity and fluid drainage capabilities of the TM. These metabolic processes play essential roles in regulating actin cytoskeletal dynamics and ECM turnover, which are necessary for proper AH outflow regulation [29–31]. These findings suggest that disruptions in glucose or lipid pathways may individually or collectively contribute to TM dysfunction and elevated IOP. Despite these insights, the specific molecular responses of TM cells to hyperglycemic conditions are not well-characterized.

Limited studies have shown that high glucose exposure can induce fibrosis in TM cells [23, 24]. Considering the important role of TM as the primary regulator of AH outflow, understanding its transcriptional responses under hyperglycemic conditions is crucial. This study aims to investigate the effects of high glucose on TM, focusing on gene expression changes that may reveal potential therapeutic targets to manage glaucoma associated with diabetes.

## Materials and methods

### Primary trabecular meshwork cell culture

Primary human TM (HTM) cells were cultured from TM tissue isolated from donor corneal rings after they had been used for corneal transplantation at the Indiana University Clinical Service, Indianapolis [32]. HIPPA compliance guidelines were followed for the use of human tissues. The usage of donor tissues was exempt from DHHS regulation and IRB protocol (1911117637), approved by the Indiana University School of Medicine IRB review board. The eye bank that provided the corneal rims also supplied information on the donors’ age, race, and sex. From the cornea, TM tissue was carefully extracted and chopped into fine pieces and placed in a 2% gelatin-coated 6-well tissue culture plated and held in place with a coverslip. The tissues were grown in OptiMEM (Gibco, #31985-070), containing 20% FBS and penicillin-streptomycin-glutamine solution (Gibco, #10378-016). The expanded population of HTM cells was sub-cultured after 1–2 weeks in DMEM, containing 10% FBS and characterized by the detection of dexamethasone-induced myocilin using rabbit anti-myocilin antibody (provided by Dr. Daniel Stamer, Duke University).

### Culturing HTM in normoglycemic and hyperglycemic conditions

Glucose-containing media was made by the addition of D-Glucose (ThermoFisher, #D161) to glucose-free DMEM (Gibco, #11966-025). Based on previous studies [24, 33], concentrations of 5.5 mM representing normoglycemic and 30 mM for hyperglycemic conditions were used for the treatment of HTM. Upon cell confluency, HTM cells from three biological replicate cells were treated with DMEM containing 5.5 mM glucose before the start of treatment simulating the normoglycemic conditions. The next day, cells were treated with fresh DMEM with either 5.5 mM or 30 mM glucose. Samples were left in these culturing conditions for one week to allow time for cells to respond to their environment.

### mRNA sequencing

#### RNA sample Preparation

Post-seven-day treatment, cells were washed with 1X PBS and collected and homogenized in TRI reagent. The total RNA was extracted and purified using Direct-zol^™^ RNA MiniPrep kit (R2050S) following the manufacturer’s protocol.

#### KAPA mRNA and hyperprep method for mRNA sequencing

Purified total RNA was first evaluated for its quantity and quality using Agilent Bioanalyzer 2100. For RNA quality, a RIN number of 7 or higher was utilized. 100ng of total RNA were used to prepare a cDNA library that includes mRNA purification/enrichment, RNA fragmentation, cDNA synthesis, ligation of index adaptors, and RNA amplification by following the KAPA mRNA Hyper Prep Kit Technical Data Sheet, KR1352-V4.17 (Roche Diagnostics, Basal, Switzerland). Resulting indexed library was quantified, quality assessed by Qubit and Agilent Bioanalyzer, and multiple libraries were pooled in equal molarity. The pooled libraries were then denatured and neutralized before loading to the NovaSeq 6000 squencer at 300 pM final concentration for 100 b paired-end sequencing (Illumina, Inc.). Approximately 30–40 M reads per library were generated. A Phred quality score (Q score) was used to measure the sequencing quality. More than 90% of the sequencing reads reached Q30 (99.9% base call accuracy).

#### Mapping QC and data analysis

The sequencing data were first assessed using FastQC (Babraham Bioinformatics, Cambridge, United Kingdom) for quality control. Then, all sequenced libraries were mapped to the human genome (hg38) using STAR RNA-seq aligner (v.2.5) [34] with the following parameter: “--outSAMmapqUnique 60.” The reads distribution across the genome was assessed using bamutils (from NGSUtils v.0.5.9) [35]. Uniquely mapped sequencing reads were assigned to hg38 refGene genes using feature Counts (from subread v.1.5.1) [36] with the following parameters: “-s 2–p–Q 10.” Differential expression analysis was performed using edgeR [37, 38]. Counts were normalized to counts per million reads (CPM) for each sample. Data were examined by Multidimensional Scaling in the edgeR package [37] to detect outliers. The data were normalized using the TMM (trimmed mean of M values) method. False discovery rates (FDR) were calculated using the Benjamini & Hochberg method within edgeR. All raw and processed data are available *via* Gene expression omnibus (GEO).

The GEO accession: GSE275629.

### Gene expression analysis

The remaining mRNA sequencing samples were used for confirmatory analysis through qPCR, where the top five most upregulated and downregulated genes were validated. Equal amounts of RNA were reverse transcribed to complementary DNA (cDNA) using the 5X All-In-One RT MasterMix (Applied Biological Materials Inc., #G492) with genomic DNA removal according to the manufacturer’s instructions. The following reaction conditions were maintained for cDNA conversion: incubation at 25 °C for 10 min, followed by incubation at 42 °C for 15 min, and enzyme inactivation at 85 °C for 5 min. The cDNA was diluted as per requirement and used for gene expression analysis using Quant Studio Flex 6 thermocycler (ThermoFisher Scientific). Bright Green 2X qPCR MasterMix-ROX (Applied Biological Materials Inc., #MasterMix-LR-XL) and gene-specific oligonucleotides (Integrated DNA Technologies) were used for the analysis. Sequence-specific forward and reverse oligonucleotide primers for the indicated genes are provided in Table 1.

Each sample for the PCR reaction was performed in triplicate using the following protocol: initial denaturation for 95 °C for 2 min followed by 40 cycles of denaturation at 95 °C for 15 s, annealing at 60 °C for 15 s, and extension at 72 °C for 1 min. An extended step was used to measure the melting curves obtained immediately after amplification by increasing the temperature in 0.4 °C increments from 65 °C for 85 cycles of 10 s each. The fold difference in the expression of test genes between the control and treatment was calculated by the delta-delta Ct method. Normalization was performed using glyceraldehyde-3-phosphate dehydrogenase (GAPDH).

**Table 1:**
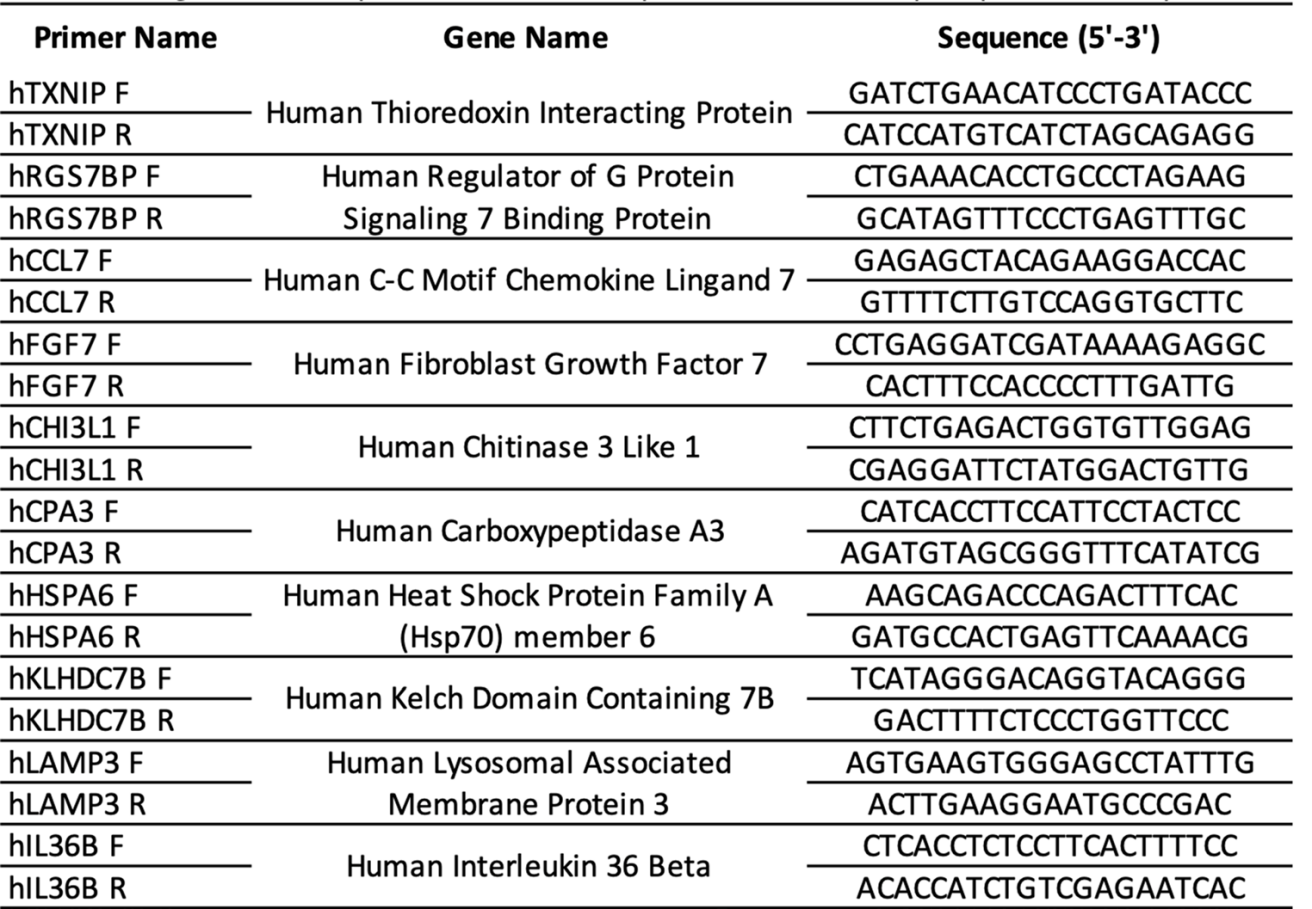
qPCR primer details

### Immunofluorescence

Immunofluorescence was performed to study the impact of glucose levels on the distribution of ECM proteins Collagen 1 and Fibronectin in HTM cells grown on 2% gelatin-coated glass coverslip culture plates under high and optimal glucose levels, as mentioned above, using published protocol from the lab [39].

### Image analysis quantification

Collagen 1 and fibronectin fluorescence intensities were quantified using CellProfiler v4.2.7. Before analysis, fluorescent images from two HTM lines (*n* = 15–16) were converted to 8-bit grayscale using ImageJ. The processed images were then imported into CellProfiler, where the Measure Image Intensity module was used to calculate the overall signal intensity.

### String network analysis

String network analysis was performed to predict the interactors for up and downregulated genes. Each network is based on no more than five interactors with line colors based on the types of interactions.

### Statistical analysis

All data are presented as the mean ± standard error of the mean (SEM) of biological replicates of three independent observations for RNA-seq and confirmatory analysis using qPCR. GraphPad Prism 8 was used to generate graphs. Data was analyzed using paired or unpaired student’s t-test. p-value ≤ 0.05 was considered statistically significant.

## Results

### RNA sequencing

**Fig. 1:**
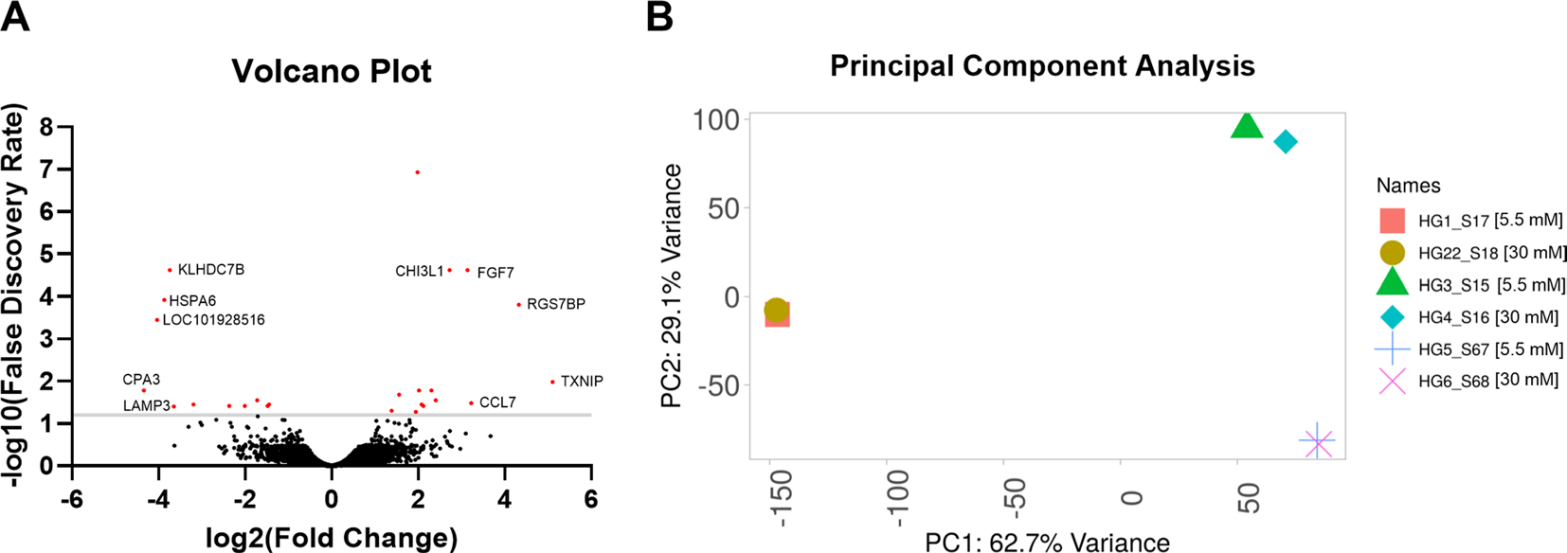
**A**) Volcano Plot showing the significantly up and downregulated genes. Top 5 upregulated and downregulated genes are marked. **B**) Principal component analysis showing the variance in loaded samples. 5.5 mM representing normoglycemic and 30 mM for hyperglycemic conditions

Primary HTM cells from three biological replicates were treated with 5.5 mM and 30 mM high glucose concentrations for seven days to evaluate the effect of high glucose on the transcriptome profile in the TM cells. An unbiased mRNA sequencing analysis identified 14,766 transcripts. Based on the statistical significance of (*p* ≤ 0.05), a false discovery rate (FDR) < 6% and log2 fold change (log2FC) of +/- 0.3, 25 genes were identified to be differentially expressed. The overall distribution of gene expression changes is visualized in the volcano plot (Fig. 1A), where red dots represent significantly altered genes (FDR < 6%, represented by the solid gray line), and black dots indicate genes with no significant change compared to controls. The top five upregulated and downregulated genes are highlighted in Fig. 1A. Principal component analysis (PCA) shows the variance in loaded samples (Fig. 1B) [40].

Among differentially expressed genes, 14 were upregulated (≥ 0.3 log2FC), and 11 genes were downregulated (≤ 0.3 log2FC) under high glucose treatment in TM cells (Table 2). Thioredoxin-interacting protein (*TXNIP*) was the topmost significantly upregulated gene (FC = 34.36; log2FC = 5.1) under hyperglycemic conditions. TXNIP is a multifunctional protein known to be involved in regulating glucose metabolism [41, 42], mediating oxidative stress [43, 44], autophagy, and apoptosis [45–47]. Besides *TXNIP*, genes that are associated with glucose metabolism- growth factor 7 (*FGF7*) (FC = 8.8; log_2_FC = 8.8), chitinase 3 like 1 (*CHI3L1*) (FC = 6.61; log_2_FC = 2.7), secretory leukocyte peptidase inhibitor (*SLPI*) (FC = 5.29; log_2_FC = 2.40), arrestin domain containing 4 (*ARRDC4*) (FC = 3.95; log_2_FC = 1.98) and snail family transcriptional repressor 1 (*SNAI1*) (FC = 3.84; log_2_FC = 1.94) were all upregulated significantly. In addition to glucose metabolism, FGF7, CHI3L1, and SNAI1 are further implicated in the epithelial- or endothelial- to mesenchymal transition (EMT/EndMT) [48–51].

**Table 2:** Genes upregulated in TM subjected to hyperglycemic conditions

*Gene Symbol*	*Accession*	*Description*	*Fold Change*	*log*_*2*_ *FC*	*p* *-value*
TXNIP	10628	thioredoxin interacting protein	34.363	5.103	5.60E-06
RGS7BP	401190	regulator of G protein signaling 7 binding protein	20.036	4.325	6.29E-08
CCL7	6354	C-C motif chemokine ligand 7	9.353	3.225	3.34E-05
FGF7	2252	fibroblast growth factor 7	8.801	3.138	6.59E-09
CHI3L1	1116	chitinase 3 like 1	6.607	2.724	5.65E-09
SLPI	6590	secretory leukocyte peptidase inhibitor	5.288	2.403	2.66E-05
GNA14	9630	G protein subunit alpha 14	4.930	2.302	1.23E-05
KRT19	3880	keratin 19	4.335	2.116	5.53E-05
STXBP6	29091	syntaxin binding protein 6	4.225	2.079	4.14E-05
KRT81	3887	keratin 81	4.052	2.019	1.18E-05
ARRDC4	91947	arrestin domain containing 4	3.947	1.981	7.96E-12
SNAI1	6615	snail family transcriptional repressor 1	3.842	1.942	5.25E-02
TENM2	57451	teneurin transmembrane protein 2	2.943	1.557	1.69E-05
COLEC12	81035	collectin subfamily member 12	2.605	1.381	5.01E-02

Genes associated with G-protein signaling, including regulator of G protein signaling 7 binding protein (*RGS7BP*) (FC = 20.03; log_2_FC = 4.33) and G protein subunit alpha 14 (*GNA14*) (FC = 4.93; log_2_FC = 2.30), were significantly upregulated, potentially influencing actin cytoskeleton dynamics through downstream signaling pathways. Additionally, keratin proteins- Keratin 19 (*KRT19*) (FC = 4.34; log_2_FC = 2.12) and Keratin 81 (*KRT81*) (FC = 4.05; log_2_FC = 2.02), which are associated with the intermediate filament structure of the cytoskeleton, were also increased. Apart from TXNIP, syntaxin binding protein 6 (*STXBP6*) (FC = 4.22; log_2_FC = 2.079), which is also involved in autophagy [52] was significantly increased.

Interestingly, from our list of upregulated genes, several are associated with immune function, notably C-C motif chemokine ligand 7 (*CCL7*) (FC = 9.35; log_2_FC = 3.23), a chemokine involved in immune cell recruitment and inflammation [53]. Other immune-related genes include *CHI3L1* and *SLPI*, which are involved in inflammatory responses [54, 55].

Among downregulated genes (Table 3), mast cell carboxypeptidase A (*CPA3*) was the most significantly downregulated (FC=-20.25; log_2_FC=-4.34). CPA3 is a member of the zinc metalloprotease family and has been implicated in the regulation of the ECM during tissue remodeling [56]. Interestingly the second most downregulated transcript is of uncharacterized protein-coding RNA (*LOC101928516*) (FC=-16.44; log_2_FC=-4.04). Transcripts of proteins involved in cellular quality control such as heat shock protein Family A (Hsp70) Member 6 (*HSPA6*) or HSP70B′ (FC=-14.60; log_2_FC=-3.87) a stress response chaperone protein; lysosome-associated membrane protein 3 (*LAMP3*) (FC=-12.56; log_2_FC=-3.651) which contributes to protein degradation and cell survival [57] and cystinosin, lysosomal cystine transporter (*CTNS*) (FC=-2.79; log_2_FC=-1.48) which helps in maintaining cellular redox homeostasis [58]; stromal cell-derived factor 2 like 1 (*SDF2L1*) (FC=-5.17; log_2_FC=-2.37) which is an endoplasmic reticulum (ER) protein involved in the prevention of protein aggregation [59]; and cyclin-dependent kinase 2-associated protein 2 (*CDK2AP2*) (FC=-2.74; log_2_FC=-1.45) involved in cell survival [60], were all downregulated significantly. High glucose treatment has also been found to decrease kelch domain containing 7B (*KLHDC7B*) mRNA levels (FC=-13.42; log_2_FC=-3.75), which is a mediator of ER stress [61]. Pro-inflammatory cytokine interleukin 36 beta (*IL36B*) (FC=-9.16; log_2_FC=-3.195) and interleukin 20 receptor subunit beta (*IL20RB*) (FC=-3.30; log_2_FC=-1.72) which is a receptor for interleukins IL19, IL20 and IL24 were reduced significantly. Occludin (*OCLN*) (FC=-4.03; log_2_FC=-2.01), a tight junction membrane protein was also significantly downregulated.

**Table 3:** Genes downregulated in TM subjected to hyperglycemic conditions

Gene Symbol	Accession	Description	Fold Change	log_2_FC	*p*-value
K2AP2	10,263	cyclin dependent kinase 2 associated protein 2	-2.735	-1.452	4.32E-05
CTNS	1497	cystinosin, lysosomal cystine transporter	-2.795	-1.483	5.70E-05
IL20RB	53,833	interleukin 20 receptor subunit beta	-3.303	-1.724	2.65E-05
OCLN	100,506,658	occludin	-4.028	-2.010	5.02E-05
SDF2L1	23,753	stromal cell derived factor 2 like 1	-5.167	-2.369	5.36E-05
IL36B	27,177	interleukin 36 beta	-9.156	-3.195	3.93E-05
LAMP3	27,074	lysosomal associated membrane protein 3	-12.563	-3.651	6.19E-05
KLHDC7B	113,730	kelch domain containing 7B	-13.418	-3.746	4.07E-09
HSPA6	3310	heat shock protein family A (Hsp70) member 6	-14.603	-3.868	4.06E-08
LOC101928516	101,928,516	uncharacterized LOC101928516	-16.440	-4.039	1.68E-07
CPA3	1359	carboxypeptidase A3	-20.252	-4.340	1.10E-05

### Confirmatory analysis for mRNA-seq data by qPCR

**Fig. 2:**
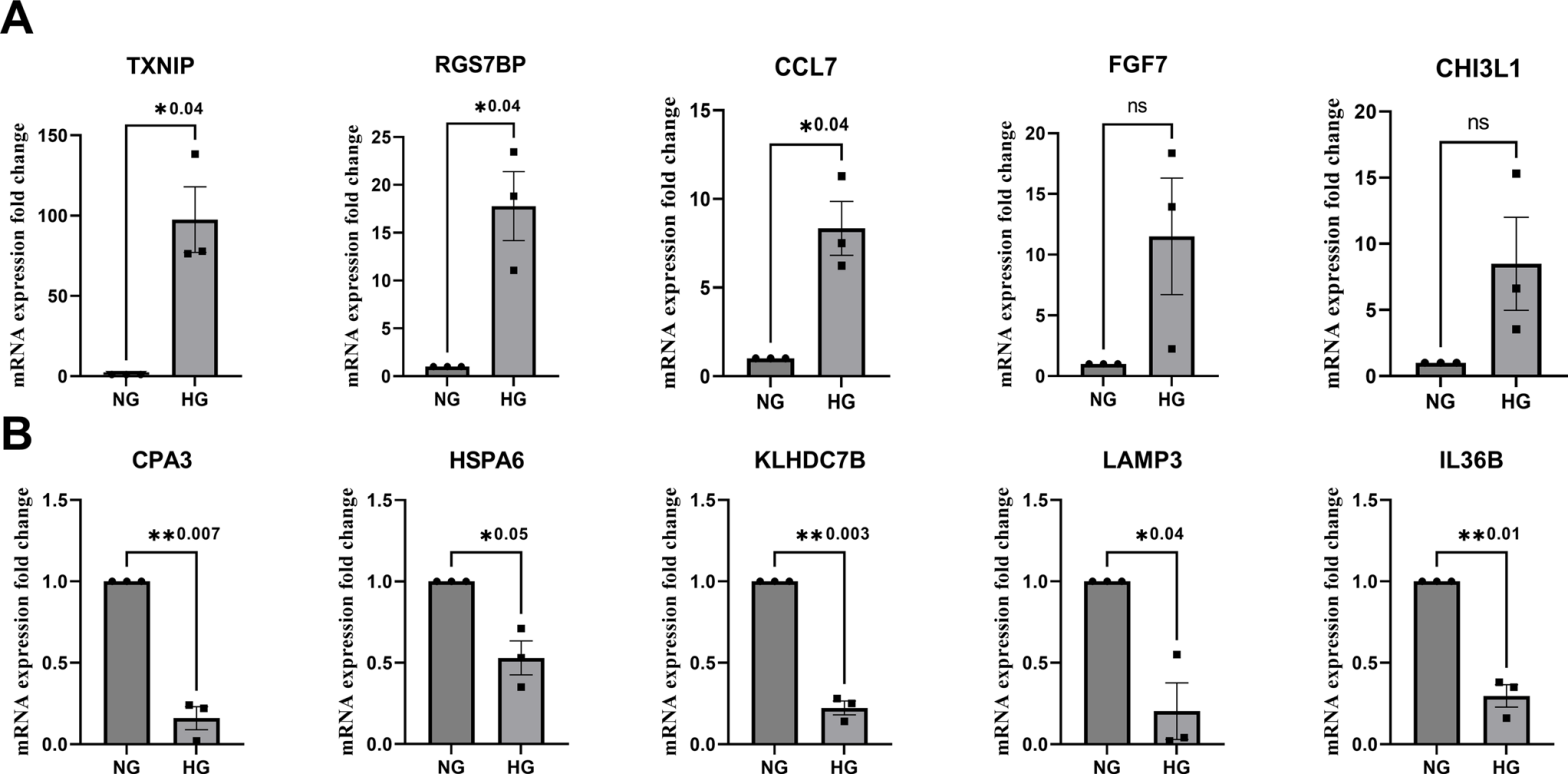
Gene expression analysis showing the top 5 up and downregulated genes. Values represent the mean ± SEM, where *n* = 3 (biological replicates). * *p* ≤ 0.05 was considered significant

Confirmatory analysis was carried out using qPCR for the top five upregulated and downregulated genes (Fig. 2) under high glucose conditions. Among the upregulated genes that were tested in qPCR, *TXNIP*, *RGS7BP*, and *CCL7* showed significant changes in mRNA expression (*p* = 0.04). Though *FGF7* and *CHI3L1* were not significant, they showed trends of upregulation. Among the genes downregulated, *CPA3* (*p* = 0.007), *HSPA6* (*p* = 0.05), *KLHDC7B* (*p* = 0.003), *LAMP3* (*p* = 0.04), and *IL36B* (*p* = 0.01) showed a significant reduction in mRNA expression fold change which was consistent with the significant changes seen for these genes in mRNA sequencing analysis.

### Predicted protein interaction analysis for differentially expressed genes

**Fig. 3:**
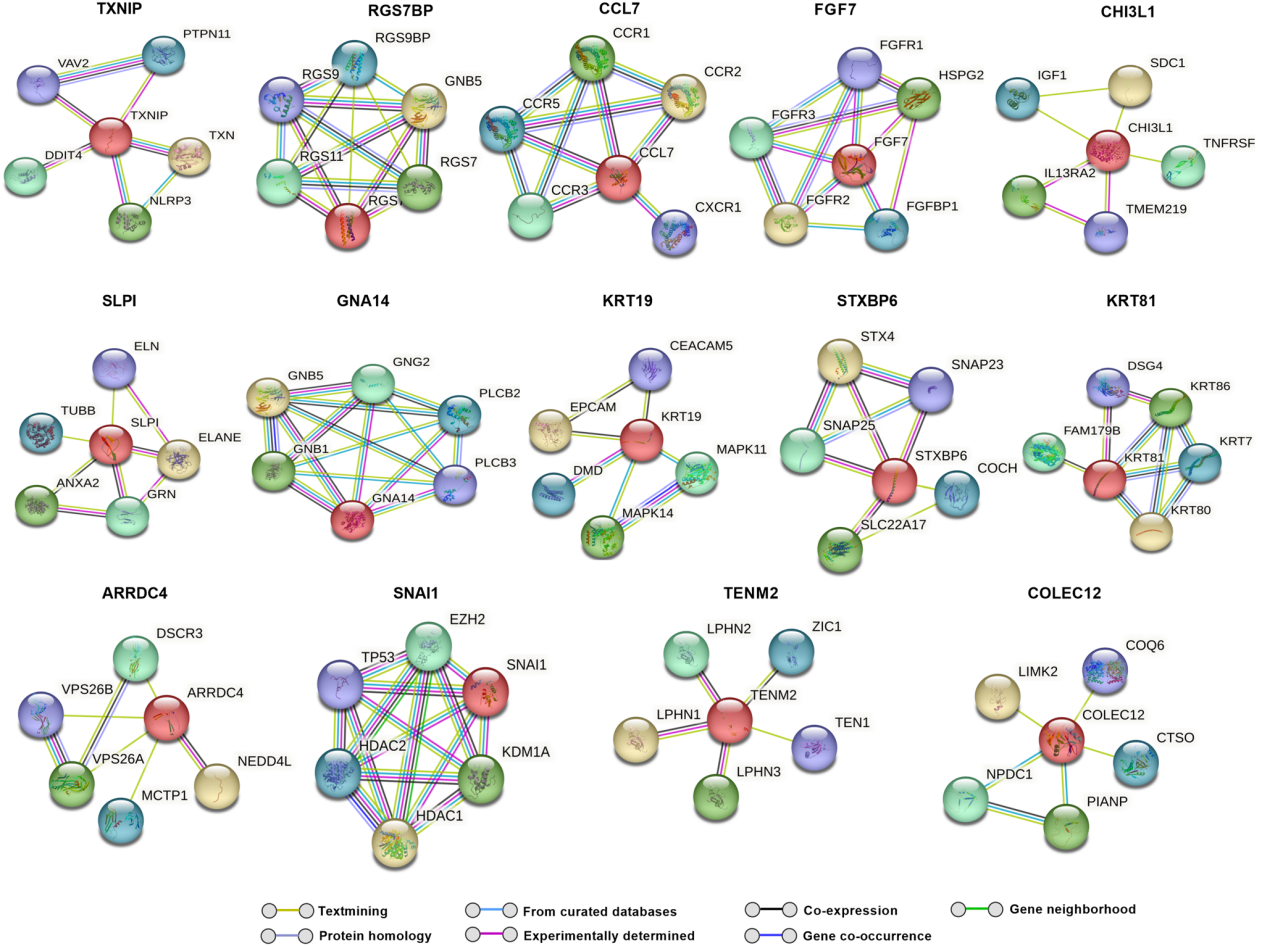
Predicted string network analysis for upregulated genes with maximum of five interactors for each gene. Lines drawn between genes indicate their interconnection

**Fig. 4:**
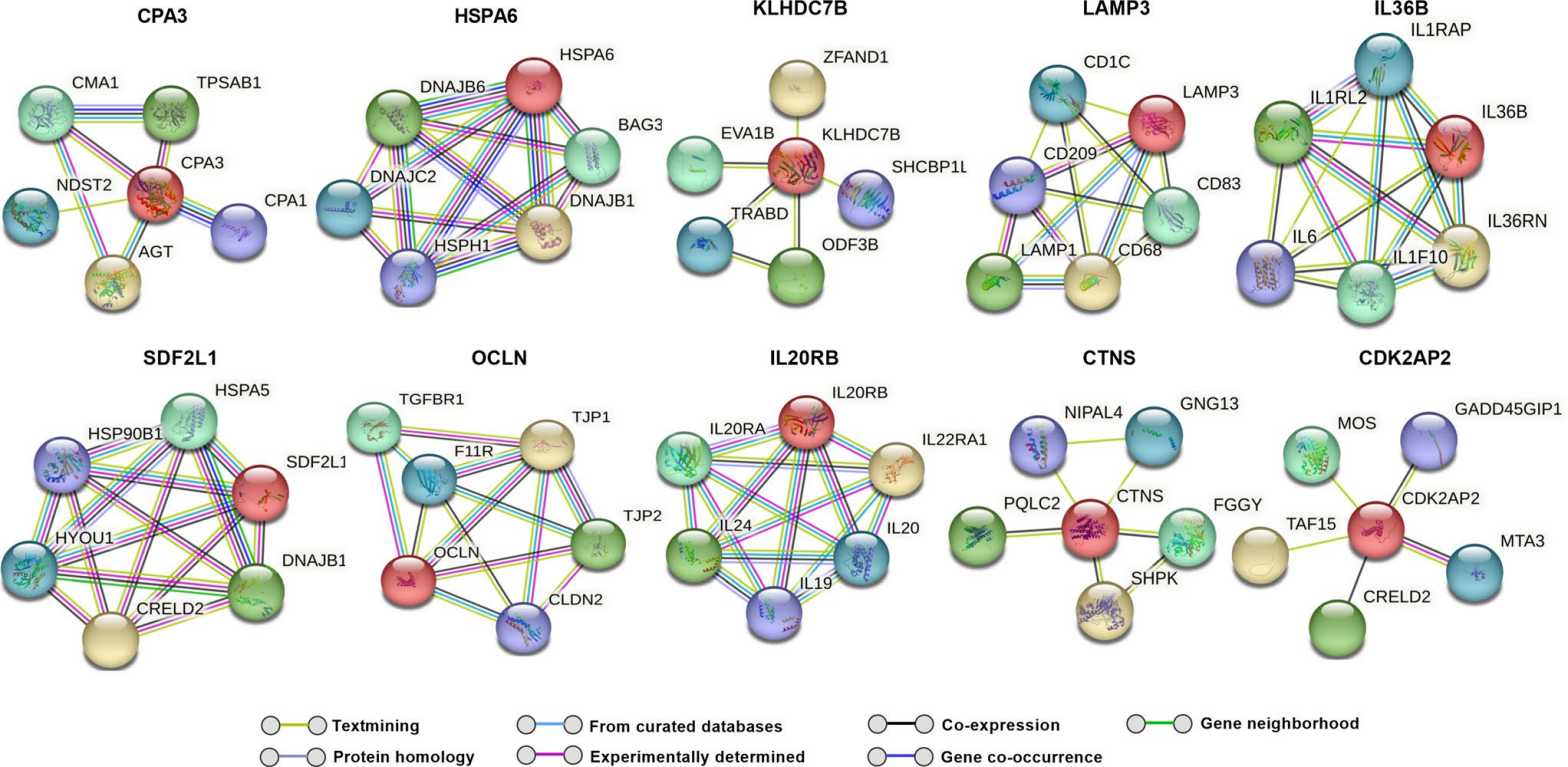
Predicted string network analysis for downregulated genes with a maximum of five interactors for each gene. Lines drawn between genes indicate their interconnection

To explore potential molecular interactions among differentially expressed genes, we performed a STRING network analysis for up and downregulated genes, which predicted several possible interactions at the protein level. This analysis, with a maximum of five immediate interactors, highlights clusters potentially associated with oxidative stress, autophagy, protein quality control, and fibrosis (Figs. 3 and 4).

**Fig. 5:**
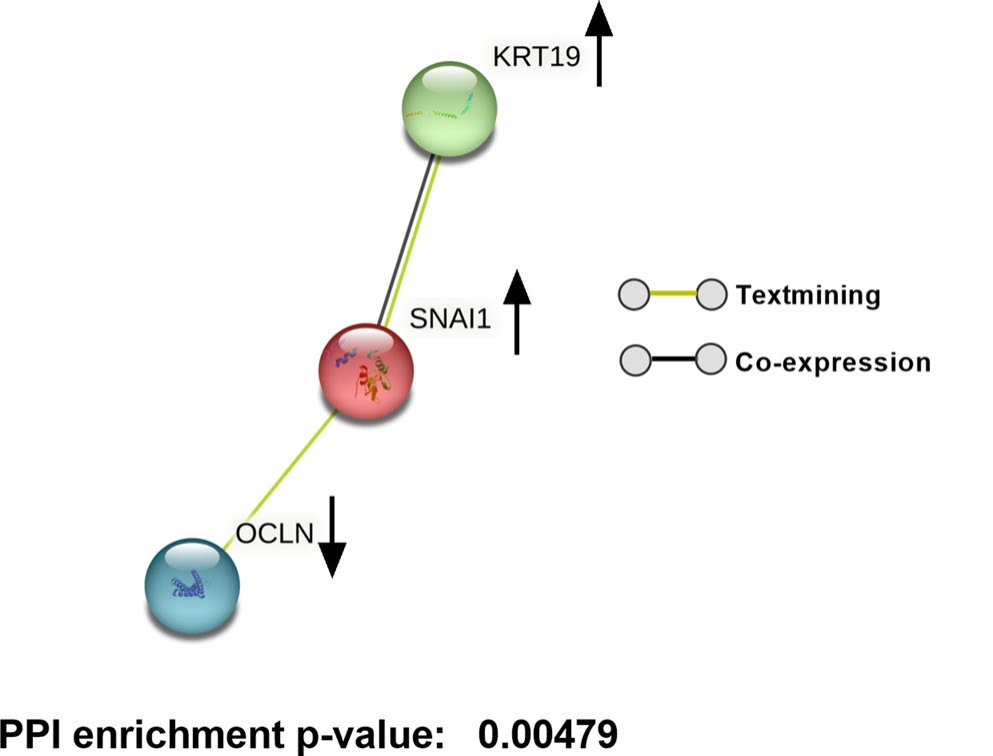
String network analysis for SNAI1, KRT19, and OCLN. Lines drawn between genes indicate their interconnection. Black arrows indicate whether the gene is up or downregulated

Notably, TXNIP showed potential interactions with DNA damage-inducible transcript 4 protein (DDIT4), suggesting a possible pathway by which high glucose-induced TXNIP upregulation could influence cellular stress responses [62]. Additionally, CHI3L1 was predicted to interact with tumor necrosis factor receptor superfamily member 10 A (TNFRSF10A) and Transmembrane Protein 219 (TMEM219), which are involved in apoptotic pathways [63, 64]. Interactors of ARRDC4, including neural precursor cells expressed developmentally down-regulated protein 4-like (NEDD4L), suggest an attempt by TM cells to counteract oxidative stress through enhanced autophagy [65]. TEN1, a possible interactor of TENM2, plays a role in DNA replication and telomere protection under stress, potentially aiding in cellular survival [66]. Additionally, we observed interactions involving KRT19, SNAI1, and OCLN (Fig. 5) that suggest a potential association of hyperglycemia with fibrosis-related pathways in TM.

### Effect of high glucose on ECM components

**Fig. 6:**
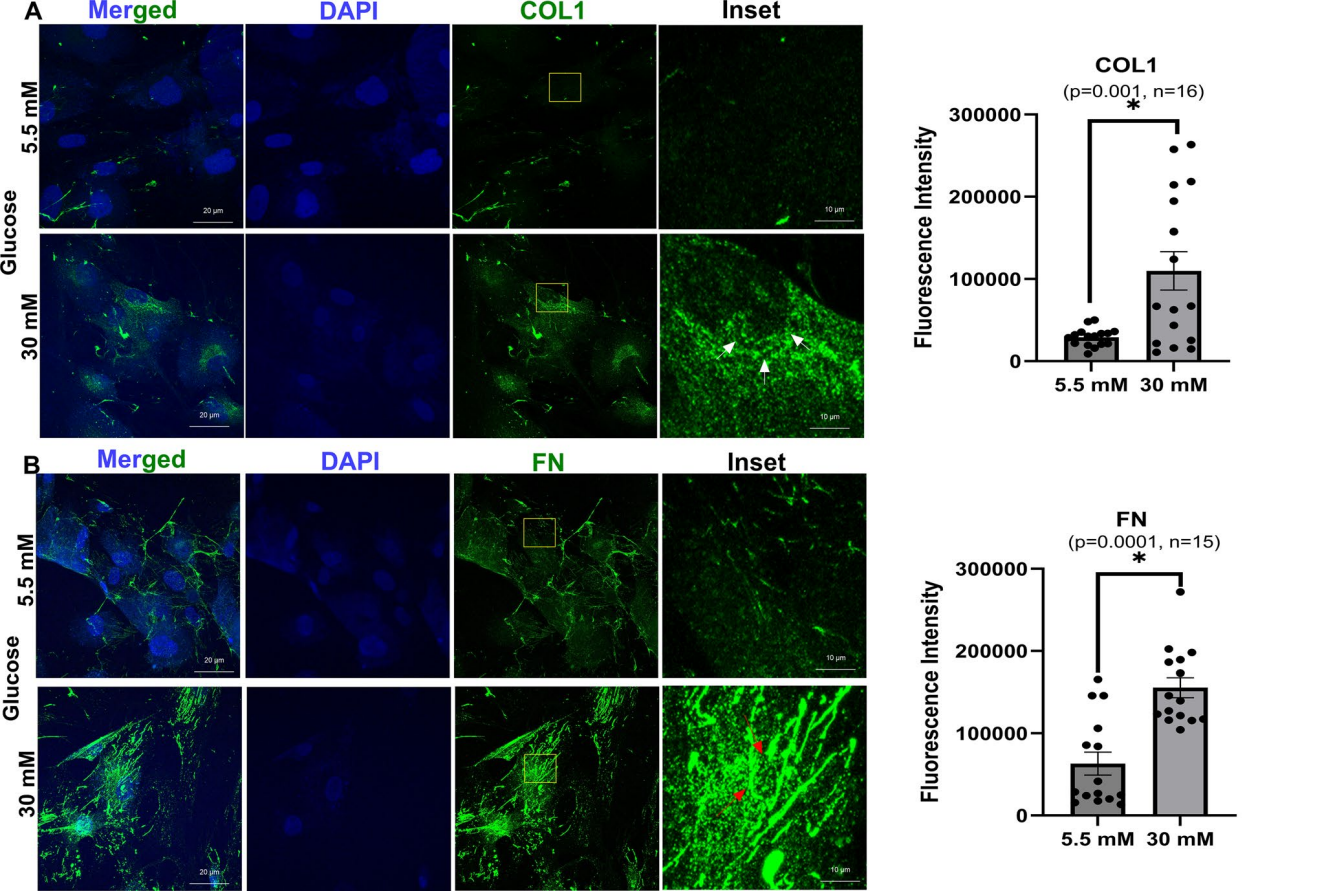
Effect of high glucose on the qualitative and quantitative cellular distribution of ECM components. **a**) Immunofluorescence staining of collagen 1 (COL1) distribution, **b**) Immunofluorescence staining of fibronectin (FN) distribution. Unpaired student’s t-test with sample size *n* = 15–16

Immunofluorescence analysis demonstrated an increased ECM distribution in HTM cells (Fig. 6) treated under high glucose (30 mM) compared to cells grown in normoglycemic conditions (control). For better visualization, the representative insets are shown in normoglycemic and hyperglycemic conditions. Increased punctate distribution of collagen 1 around the perinuclear region (indicated by white arrows) (Fig. 6A), as well as fibrillar staining (indicated by red arrows) for fibronectin (Fig. 6B) in hyperglycemic conditions, were seen in hyperglycemia. Up on quantifying the intensities of the immunopositive staining of collagen 1 and fibronectin, we found a significant increase in collagen 1 (*p* = 0.001, *n* = 16) and fibronectin (0.0001, *n* = 15).

## Discussion

This study provides the first comprehensive insight into the effects of high glucose on gene expression in HTM cells in an unbiased approach, highlighting potential mechanisms by which hyperglycemia contributes to glaucoma pathogenesis. Our findings reveal significant transcriptional changes, with key upregulated genes associated with oxidative stress, apoptosis, autophagy, immune response, and fibrosis, processes that play critical roles in TM function and IOP regulation under hyperglycemic conditions.

High glucose exposure in TM cells induces oxidative stress, triggering multiple pathways that impact cellular survival, autophagy, and protein quality control, ultimately compromising TM function. One of the most significantly upregulated genes in our dataset was *TXNIP*, a known regulator of oxidative stress and apoptosis. TXNIP induces reactive oxygen species (ROS) production, leading to cellular oxidative stress and activation of apoptotic pathways, such as the MAPK pathway [43, 47]. This effect is consistent with previous studies linking TXNIP with glucose metabolism dysregulation and oxidative stress in diabetic conditions [67]. While the role of the insulin signaling pathway in TM is not well established, TXNIP is known to antagonize insulin signaling in other cell types affecting glucose uptake [67–69].

High glucose-induced oxidative stress also appears to disrupt autophagy and protein quality control, processes essential for cellular homeostasis in TM cells. Our data show the downregulation of autophagy-associated genes *HSPA6* and *LAMP3*. HSPA6 functions as a heat shock protein involved in protein quality control under stress conditions [70], and its downregulation could compromise cellular resilience to high glucose-induced stress. Additionally, LAMP3, an autophagy inhibitor, was also downregulated, potentially indicating impaired autophagic flux [71], which could lead to the accumulation of damaged proteins and organelles. Furthermore, KLHDC7B, an ER stress mediator, was downregulated, suggesting additional impairment in protein folding and degradation mechanisms [61]. Compromised protein quality control and disrupted autophagy in TM can lead to increased cellular stress, contributing to TM stiffness and reduced AH outflow [72–74].

Interestingly, our data suggests that high glucose can trigger immune and inflammatory responses in TM cells. The upregulation of immune-related genes, such as *CCL7*, *CHI3L1*, and *SLPI*, suggests an inflammatory response. *CCL7* is a chemokine involved in immune cell recruitment, suggesting that inflammatory processes are activated by high glucose exposure in TM cells. Previous studies have shown upregulation of CCL7 gene expression in TM in response to mechanical stretch and models of controlled IOP elevation [73, 75]. *CHI3L1* is one of the more abundant transcripts in TM cells [76] and can promote inflammation by stimulating the production of pro-inflammatory cytokines like IL-6 and TNF-α [77, 78]. Upregulation of the anti-inflammatory gene *SLPI* [79] is likely a compensatory response to mitigate excessive inflammation. Chronic inflammation in TM cells can trigger ECM remodeling, leading to fibrosis and potentially compromising AH outflow [80, 81].

A significant finding in our study is the involvement of genes linked to ECM remodeling and fibrosis, such as *SNAI1*, *KRT19*, *FGF7*, and *CHI3L1*. SNAI1, a transcription factor promoting fibrosis [48], is upregulated in response to high glucose treatment. FGF7, and CHI3L1 which are reported to be involved in EMT [49–51] were upregulated. KRT19, associated with the Wnt/β-catenin signaling pathway, has been implicated in fibrotic processes and contributes to TM stiffness and dysfunction [82–84]. Our findings align with previous studies that have shown high glucose can induce fibrosis in TM [23, 24]. Protein interaction analysis indicates SNAI1 and KRT19 interaction at the protein level (Fig. 5), driving fibrotic pathways in the TM under hyperglycemic conditions. CPA3, a serine carboxypeptidase that cleaves C-terminal basic amino acids from ECM components like collagen and fibronectin, plays a role in ECM remodeling. Downregulation of CPA3 under high glucose conditions potentially contributes to ECM accumulation. The increased stiffness and ECM accumulation associated with fibrosis can increase resistance to AH outflow, contributing to elevated IOP and the risk of glaucoma in diabetic patients. This data strongly indicates that under hyperglycemic conditions, the expression, secretion, and fibril formation of ECM can increase. The altered distribution of Collagen 1 and fibronectin as demonstrated by the qualitative and quantitative increase suggests a direct impact of hyperglycemia on the structural aspects of TM. This also aligns with an earlier published report [24]. Despite donor variability, high glucose-induced targeted gene expression changes (oxidative stress, fibrosis, immune response) in TM cells, rather than global shifts, reflect the gradual nature of diabetic glaucoma pathology.

While the current study focuses on glucose dysregulation, our previous research demonstrated that lipid metabolism, including *de novo* lipogenesis and cholesterol regulation, plays a critical role in regulating actin cytoskeletal dynamics and maintaining AH outflow [29, 30]. Together, these findings highlight the complex metabolic responses in TM cells and suggest that disruptions in glucose and/or lipid metabolism can lead to TM dysfunction. Further studies are needed to explore the interplay between glucose and lipid metabolic pathways under hyperglycemic conditions and their implications for therapeutic targeting.

Our STRING analysis predicted several protein-protein interactions, providing valuable insights into potential pathways impacted by high glucose. However, these findings are based on predictive modeling and require further experimental validation. Future studies are needed to validate these pathways, ideally through protein-level analyses, functional assays, and in vivo studies to determine their exact roles in TM dysfunction and glaucoma pathogenesis under hyperglycemic conditions.

In conclusion, this study demonstrates that high glucose exposure in TM cells induces multiple stress-related pathways, including oxidative stress, apoptosis, immune response, autophagy dysregulation, and ECM remodeling. These findings suggest that chronic hyperglycemia in diabetic patients could exacerbate TM dysfunction, leading to impaired AH outflow and increased IOP, a known risk factor for glaucoma. Targeting pathways involved in oxidative stress and fibrosis, such as TXNIP, SNAI1, and related signaling pathways, can provide new therapeutic approaches in managing elevated IOP and mitigating glaucoma progression in diabetic patients.

Although this study provides valuable transcriptomic insights and changes in ECM remodeling in vitro in TM cells, there are multiple limitations to this study. Future studies utilizing in vivo models or ex vivo anterior segment perfusion systems will be necessary to confirm the physiological relevance of these findings and their impact on aqueous humor outflow and IOP regulation. Further studies are warranted to investigate the long-term impact of high glucose on TM cells and to explore potential interventions that could restore TM function, reduce ECM accumulation, and modulate oxidative stress in the context of diabetes-related glaucoma. This study in in vitro model does not fully recapitulate the complex in vivo microenvironment, and further studies are required to help us better understand the relationship between diabetes and glaucoma at a cellular level as the TM cells in vivo are influenced by additional factors such as aqueous humor dynamics, mechanical forces, and systemic metabolic regulation. The role of hyperglycemia in influencing the aforementioned factors has not been studied, which can provide clues to why some individuals with hyperglycemia get glaucoma whereas others do not.

## Data Availability

Sequence data that support the findings of this study have been deposited in the Gene expression omnibus (GEO) with the primary accession code GSE275629.
